# Sex-related difference in human white matter volumes studied: Inspection of the corpus callosum and other white matter by VBM

**DOI:** 10.1038/srep39818

**Published:** 2017-01-03

**Authors:** Akihiko Shiino, Yen-wei Chen, Kenji Tanigaki, Atsushi Yamada, Piers Vigers, Toshiyuki Watanabe, Ikuo Tooyama, Ichiro Akiguchi

**Affiliations:** 1 Molecular Neuroscience Research Center, Shiga University of Medical Science, Ohtsu, Shiga, Japan; 2 College of Information Science and Engineering, Ritsumeikan University, Kusatsu, Shiga, Japan; 3 Research Institute, Shiga Medical Center, Moriyama, Shiga, Japan; 4 Biomedical Innovation Center, Shiga University of Medical Science, Ohtsu, Shiga, Japan; 5 Department of Health Science, Kyoto Koka Women’s University, Ukyo-ku, Kyoro, Japan.

## Abstract

It has been contended that any observed difference of the corpus callosum (CC) size between men and women is not sex-related but brain-size-related. A recent report, however, showed that the midsagittal CC area was significantly larger in women in 37 brain-size-matched pairs of normal young adults. Since this constituted strong evidence of sexual dimorphism and was obtained from publicly available data in OASIS, we examined volume differences within the CC and in other white matter using voxel-based morphometry (VBM). We created a three-dimensional region of interest of the CC and measured its volume. The VBM statistics were analyzed by permutation test and threshold-free cluster enhancement (TFCE) with the significance levels at FWER < 0.05. The CC volume was significantly larger in women in the same 37 brain-size-matched pairs. We found that the CC genu was the subregion showing the most significant sex-related difference. We also found that white matter in the bilateral anterior frontal regions and the left lateral white matter near to Broca’s area were larger in women, whereas there were no significant larger regions in men. Since we used brain-size-matched subjects, our results gave strong volumetric evidence of localized sexual dimorphism of white matter.

Human sexual and gender differences are of social and scientific interest. Several reports have indicated sex-related differences in form and function in human brains. In particular there has been debate about the size of the corpus callosum (CC) for more than 20 years. Accumulating evidence indicates that, on average, men have larger absolute volumes of total grey and total white matter^1^,^2^,^3^, and that the absolute size of the CC is larger in men, as would be expected on the basis of larger brain size in men^4^,^5^. However, no generally accepted protocol is apparent by which variables such as CC size should be assessed in other than absolute terms, and both the structuring of the question and the method of approach are crucial for appropriate interpretation. Smith (2005)^6^ distinguished proportionality or relative size versus statistical control for size, and suggested: “The methodological disputes that have fueled the ongoing controversy are based on misunderstandings rather than on substance, resulting in almost two decades of wasted argument”.

In discussion of CC size relative to brain size, one might use simple ratios, typically with midsagittal CC cross-sectional area (CCA) as the numerator and brain size (total intracranial volume, TIV, or some substitute indicator for volume such as brain weight) as denominator. Such ratios may be problematic. Firstly, if the dimensions chosen for the numerator (e.g., CCA) differ from those chosen for the denominator (e.g., TIV), the ratio may change in value as an object’s shape changes ^6^. Secondly, inherent in the simplicity of a simple ratio is the absence of any system to remove variance associated with the denominator, and the obvious denominator, brain size, is already known to be sexually dimorphic^7^. Several sources have emphasized that simple ratios are inappropriate for statistical control^8^,^9^,^10^,^11^, and to eliminate the variance of that covariate-denominator, statistical control is needed.

There has been dispute over interpretations of relative size difference, regardless of whether sex-related differences were found in CC size after controlling for total brain size. Various authors have contended that any observed difference is not sex-related but is due to differences in brain size^12^,^13^,^14^,^15^,^16^,^17^,^18^,^19^,^20^,^21^.

Against that background, there was an interesting study by Ardekani *et al*.^7^ that elegantly bypassed hypotheses about sizes by measuring the CC cross-sectional area in 37 TIV-matched man-woman pairs of young people^7^. The subjects of that study were extracted from the Open Access Series of Imaging Studies (OASIS) database, and CCA was measured by three-dimensional (3D) brain MRI, using a fully automatic multiatlas-based method for segmentation, adding minor manual editing in the few cases in which the automatic algorithm failed. They found that the midsagittal CCA was larger in women than in men^7^.

Since we are interested in subregional sex-related differences in the CC, and the OASIS data are in the public domain, we processed the OASIS data using voxel-based morphometry (VBM). This technique enables semi-quantitative volume evaluation of a target even in structures with unclear boundaries if regions of interest (ROI) are pre-set, therefore we created a 3D ROI mask (see Methods below for details) to measure the volume of each CC for comparison between women and men.

## Results

### Women showed larger corpus callosum volumes than men

**Figure 1:**
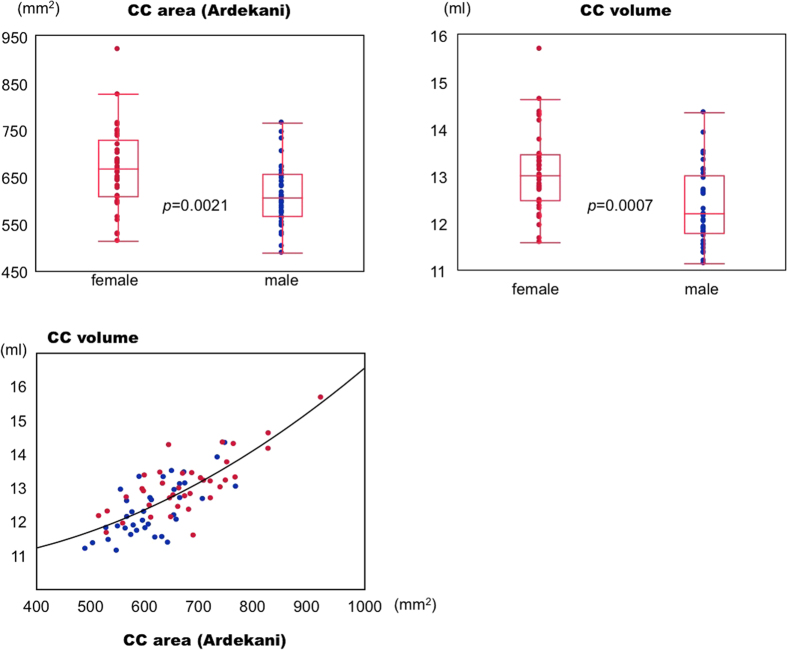
Distribution of volumes of corpus callosum (CC), among 37 man-woman pairs closely matched for age and total intracranial volume (TIV). Measurement of CC volume is performed by presetting the CC volume of interest (see Fig. 4). The upper left graph shows the distribution of the midsagittal CC area reported by Ardekani *et al*. The lower graph shows the relation between our results (CC volume) and Ardekani’s CCA findings. The *p* values are the results of Mann-Whitney U tests.

There was a statistically significant difference between men and women in the CC volumes in the 37 TIV-matched pairs. The CC volume was larger in women, with significance found by Mann-Whitney U test at *p* values of 0.0007 (Fig. 1). This result is consistent with the report by Ardekani *et al*.^7^ showing larger CCAs in women, using the same OASIS subjects whom we later studied. We found a correlation between CCA and CCV, the correlation coefficient being 0.77, better fitting a second-degree polynomial (R^2^ = 0.59) than a linear fit (R^2^ = 0.58).

### Regional sexual differences in white matter

**Figure 2:**
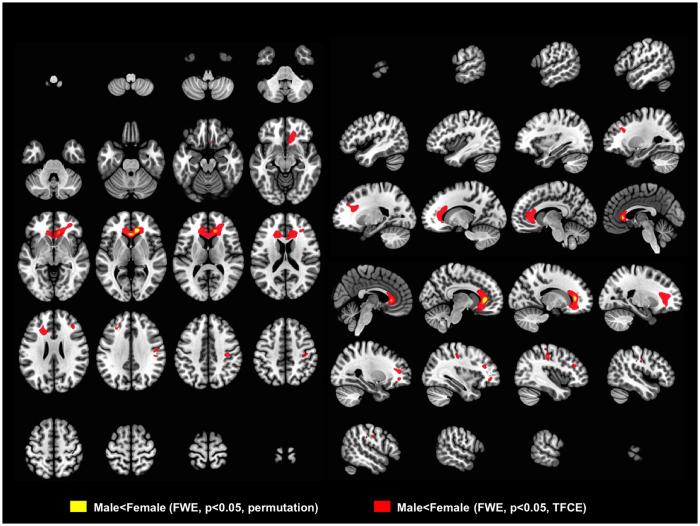
VBM results showing sex-related differences analyzed in the 37 TIV-matched man-woman pairs. Thresholds were set at familywise error (FWE) rate < 0.05 in permutation testing (yellow) and TFCE testing (red). Colored areas indicate volumes significantly larger in women than in men. As shown in this figure, the genu of the corpus callosum is significantly larger in women.

There were also statistically significant sex-related differences in those 37 TIV-matched pairs in several white matter regions when analyzed by multiple regression, treating age and TIV as confounding covariates. In the CC, the genu was the subregion showing significant sex-associated difference, being larger in women when analyzed by permutation test (FWE *p* < 0.05). Frontal white matter was larger in women, with left-side predominance in the middle frontal lobe near to Broca’s area when analyzed by threshold-free cluster enhancement (TFCE) (FWE *p* < 0.05, TFCE, Fig. 2). There was also a small cluster of significance in the primary motor cortex near to the hand and face areas, larger in women (FWE *p* < 0.05, permutation, Fig. 2).

**Figure 3:**
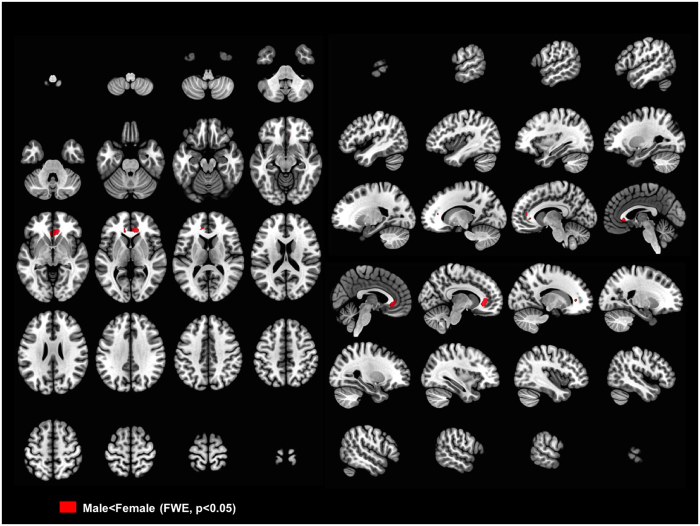
VBM results showing sex-related differences analyzed in the 37 TIV-matched man-woman pairs. Thresholds were set at familywise error (FWE) rate < 0.05. Statistical analyses were done by SPM’s RFT method. Colored areas indicate volumes significantly larger in women than in men. As shown in this figure, the genu of the corpus callosum is significantly larger in women. In FWE; FWHM = 14.6, 13.6, 13.8 mm, and 329.0 resels.

We also used SPM’s standard inference method, thresholding with Random Field Theory (RFT), and again found a significant cluster in the CC genu larger in women (FWE *p* < 0.05, FWHM = 14.6, 13.6, 13.8 mm, 329.0 resels, no small volume correction) (Fig. 3). In these analyses we found no regions in which men had larger white matter volumes than did women.

## Discussion

There have been several reports supporting the concept that the observed differences in CCA are not sex-related but brain-size-related. To settle the question, Ardekani *et al*.^7^ sorted data on young subjects from the OASIS database to match man-woman pairs very closely by TIV, to render any effect of TIV irrelevant. Then, measuring the CCA on the midsagittal plane, they found that the CCA was larger in women by a few percent on average. Since the structural boundary of the CC can only be delineated at its sagittal plane only as an area, and most such comparison studies have been area-based, we tried to apply a volumetric approach by creating a 3D CC ROI, and also analyzed the whole white matter in a voxel-by-voxel manner.

First we studied whether our VBM method could reliably show results consistent with the previous study^7^ using the same 37-pair cohort. The procedures of segmentation, warping, and smoothing in VBM may introduce some inaccuracies; for example, smoothing kernels tend to render data more normally distributed. However, the segmentation procedure is ameliorated in current VBM technology (Sup. Fig. 1), and the reliability of tissue normalization has been improved recently with the introduction of the diffeomorphic anatomical registration through exponentiated Lie algebra (DARTEL) algorithm^22^. Voxel-wise analysis allows evaluation of brain morphometric differences automatically on a whole brain basis, and it shows an advantage in studying regions that are not easily defined anatomically, such as the 3D boundary between the corpus callosum and neighboring white matter. As shown in the volumetric results, our VBM study reached reasonable concordance with the previous, area-based report, indicating larger CC volume in women within the TIV-matched man-woman pairs. On this basis, we think that VBM can evaluate morphological differences reliably enough for our purpose of finding regional and subregional sexual differences in white matter.

The salient results of our study show subregional sex differences in white matter, perhaps the most obvious being that the genu was the subregion contributing most to the CCA and CC volume sex-related differences in this TIV-matched cohort. Since the cluster of significance was found within the genu (Fig. 2), this is highly suggestive of an intrinsic morphometric sexual characteristic. Results from TFCE, an enhanced cluster-based analysis^23^, indicated that women have larger white matter volumes in the bilateral anterior frontal regions and in the primary motor cortex near to the hand and face area. There were no clear differences in white matter deep to Broca’s area (Brodmann areas 44 and 45), but white matter in the left frontal lobe, rather superior to Broca’s area was significantly larger in women (Fig. 2, Sup. Fig. 2), which region is also thought to be related to language production^24^.

Sexual dimorphism of the CC has also been the focus of some studies in diffusion tensor imaging, but to date, the results have varied. Of the reported studies, one^25^ found an increase in fractional anisotropy (FA) throughout the CC in women, and this difference was confirmed with a manually-set ROI at the genu of the CC. This study also found that men had higher FA in the cerebellum and in an area at the anterior portion of the left superior longitudinal fasciculus. Another study found that women had significantly lower regional FA values than men in the CC as a whole, but significantly higher FA values in subregions of the rostrum, genu and splenium^26^. Liu F. *et al*.^27^ measured fiber density index (FDi), that provides a relative value of the number of fiber paths per unit area. Interestingly, they found higher FA in the genu of the CC in men whereas women had a trend for increased FDi within the genu, which FDi finding qualitatively agrees with a previous post-mortem study that demonstrated higher density of axons in the CC genu in healthy women compared to that in healthy men^28^. A recent study of connection-wise statistical analysis^29^ reported that men had greater within-hemispheric connectivity whereas between-hemispheric connectivity and cross-module participation predominated in women. This study using lobar connectivity weight analysis also showed that women had higher between-hemisphere connectivity for the frontal lobes than did men. Referring to these reports, our findings of sex-linked morphometric difference in the genu of the CC may indicate that women have more interhemispheric connection in the anterior frontal lobes than do men.

Several potential limitations exist in our study. First, VBM has inherent limitations including “methodological and parameter choices in segmentation, registration, and correction for multiple comparisons, that may heavily vary and influence the results”^30^. VBM is highly sensitive to the performance of the registration and segmentation process, that is, deformation inaccuracy or misregistration may influence the results^31^,^32^. Much work has been done to alleviate such potential sources of error, including developing unified segmentation^33^ and DARTEL algorithms^34^. DARTEL was developed to improve inter-subject registration by more accurately defining a template space and innovating many more parameters to describe brain shape than any algorithm previously used in SPM. The purpose of smoothing in VBM is to reduce residual intersubject variances after normalization, though this tends to lower the sensitivity of the method due to morphological variability of the surrounding structures. Second, macroscopic morphometric differences in the CC do not directly reveal the number of fiber tracts; for example, the diameters of axons crossing through the CC vary^35^. According to a post-mortem study^36^, however, thin fibers are most dense in the genu in the human brain, therefore the larger genu volumes in women observed in our study may reflect more fiber tracts in women than in men in this subregion. Third, the sample size of this study was relatively small.

## Methods

We obtained MR images from the OASIS cross-sectional dataset, composed of normal young adult under 30 years old. All subjects were right-handed. OASIS provides freely available data and the full details can be found elsewhere^37^. The 37 TIV-matched pairs were selected as in the previous report^7^. The MR images were conducted on a 1.5 T Siemens Vision scanner (Erlangen, Germany). The volumes of the brain images were acquired by 3D T1-weighted magnetization prepared rapid gradient-echo (MPRAGE) scans. The acquisition parameters were as follows: repetition time: 9.7 ms; echo time: 4.0 ms; inversion time: 20 ms; delay time: 200 ms; and flip angle: 10°, volumes of matrix size: 256 × 256 × 128, and voxel size: 1 × 1 × 1.25 mm^3^. Each subject underwent three or four individual scans, and the images were averaged to get single, high-contrast 3D images. To make the averaged image, a 12-parameter affine transformation was computed to minimize the variance between the first MPRAGE image and the atlas target^38^. The remaining MPRAGE images were registered to the first image allowing in-plane stretch, and resampled via transform composition into a 1-mm isotropic image in atlas space.

VBM analysis was performed using Statistical Parametric Mapping (SPM) 8 software (Wellcome Trust Centre for Neuroimaging, London, UK) implemented in Matlab software (version 8.0, Mathworks Inc., Natick, MA, USA). Each brain image was first coregistered to an ICBM 152-space average template, then bias-corrected to reduce intensity inhomogeneity. The 3D head images were skull stripped and segmented into gray matter, white matter, and cerebrospinal fluid using unified segmentation as implemented in the “new segmentation” process in the SPM tool. Settings for bias correction (very light regularization), full width half maximum (FWHM, 60 mm cutoff), tissue probability maps, and affine regularization were set in the default mode. The segmented white matter images were non-linearly transformed by DARTEL to create a new template among the young adult brains, and then normalized to the MNI space with image modulation, followed by smoothing with an 8 mm FWHM kernel.

White matter regional volumes were compared between the sexes (treating sex as a dummy variable) by multiple regression analysis using SPM8. Age and TIV were regarded as nuisance variables, and so were included in the analysis as covariates of no interest. Absolute threshold masking was set at 0.1 to prevent interference by non-brain voxels. The resulting statistics were transformed to z-scores for every voxel and displayed as parametric maps using BAAD software that was developed in our laboratory (http://www.shiga-med.ac.jp/~hqbioph/BAAD (English)/BAAD.html). BAAD calculates regional brain volume (ml), through the integrated MarsBar software (http://marsbar.sourceforge.net) of which the results are independent of the masking threshold. The 3D ROI of each CC was delineated by using ITK-SNAP (http://www.itksnap.org/pmwiki/pmwiki.php) and the WFU PickAtlas software (http://fmri.wfubmc.edu/software/pickatlas) which is also integrated in BAAD, and processed with the integrated MarsBar software (Sup. Fig. 1). Results were surface-rendered using MRIcroGL software (http://www.cabiatl.com/mricrogl).

**Figure 4:**
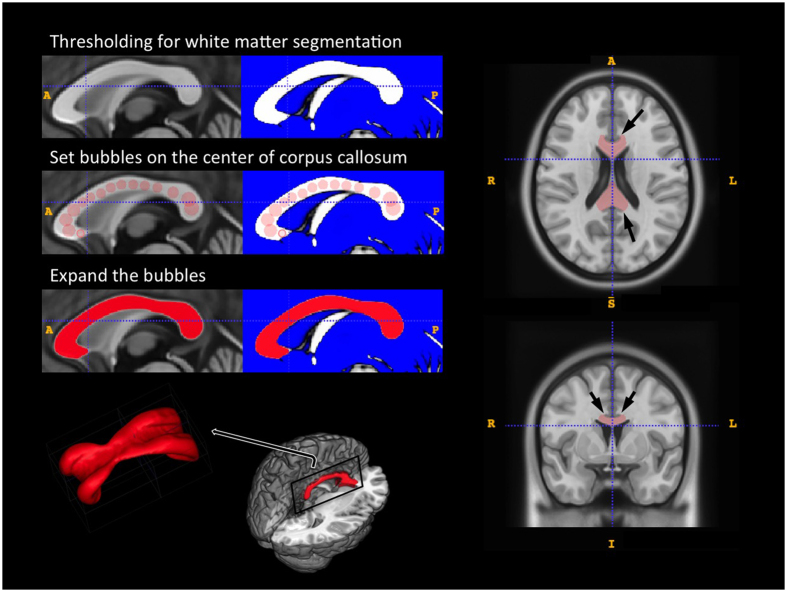
Illustration of the three-dimensional volume of interest for the corpus callosum. The ROI was made based on ICBM152 (mni_icbm152_t1) from the McConnell Brain Imaging Centre using ITK-SNAP software. We first distinguished white matter from other tissues by thresholding, then set “bubbles” on the center of the CC as the “seed points”, and expanded them after adjusting the region competition force and the curvature force parameters. The margin of the cingulate gyrus was used as a guide to set the end-point of the expansion (arrows).

We used the Montreal Neurological Institute (MNI) standard brain template, ICBM152 (mni_icbm152_t1) from the McConnell Brain Imaging Centre (http://www.bic.mni.mcgill.ca/ServicesAtlases/ICBM152NLin2009) and created a CC ROI by semi-automatic three-dimensional segmentation, using ITK-SNAP software. We first distinguished white matter from other tissues by thresholding, then set “bubbles” on the center of the CC as the “seed points”, and expanded them after adjusting the region competition force and the curvature force parameters. The basic theory of the 3D geodesic active contour method in ITK-SNAP has been described elsewhere^39^. The end-point of the expansion for the 3D boundary of the ROI was set up not to overlap the adjacent cingulate gyrus (Fig. 4). The CC volumes were measured in non-smoothed modulated images in MNI space (shown by the file name “mwc2” in SPM) by counting the total voxel volume within each ROI. The basic theory of voxel volume and partial volume effects in modulated images is well described by Radua *et al*.^40^.

The threshold for significance in the VBM analyses was set at *p* < 0.05 corrected for family-wise error (FWE) rate. For voxel-level inference, we applied permutation tests using statistical non-parametric mapping (SnPM) software (http://www2.warwick.ac.uk/fac/sci/statistics/staff/academic-research/nichols/software/snpm) by running 5000 permutations without variance smoothing. In addition to the voxel-level inference, we applied TFCE for a mixture of voxel and cluster level inference by using SPM’s TFCE toolbox (http://dbm.neuro.uni-jena.de/tfce/) developed by Dr. Gaser. The 5000 permutations were run without variance smoothing. For reference purposes, we also showed the FWE results obtained using random field theory, a widely accepted SPM inference method.

In ROI analysis, statistical significance was calculated using JMP software (version 5.01, SAS Institute Inc., NC, USA), and results yielding *p* < 0.05 were considered statistically significant (Mann-Whitney U test).

## Additional Information

**How to cite this article**: Shiino, A. *et al*. Sex-related difference in human white matter volumes studied: Inspection of the corpus callosum and other white matter by VBM. *Sci. Rep.* **7**, 39818; doi: 10.1038/srep39818 (2017).

**Publisher's note:** Springer Nature remains neutral with regard to jurisdictional claims in published maps and institutional affiliations.

## Supplementary Material

Supplementary Dataset 1
