# Supplementary material for: Sex-related difference in human white matter volumes studied: Inspection of the corpus callosum and other white matter by VBM
Source: Sci Rep. 2017 Jan 3;7:39818. doi: 10.1038/srep39818 (PMC5206615; doi:10.1038/srep39818)
Supplement: Supplementary Dataset 1 [file srep39818-s1.doc]

**Sex-related difference in human white matter volumes studied: Inspection of the corpus callosum and other white matter by VBM**

***Akihiko Shiino, ^a^ Yen-wei Chen, ^b^ Kenji Tanigaki, ^c^ Atsushi Yamada, ^d^ Piers Vigers ^a^ Toshiyuki Watanabe, ^e^ Ikuo Tooyama,^a^ and Ichiro Akiguchi ^e^**

**^a^** Molecular Neuroscience Research Center, Shiga University of Medical Science

**^b^** College of Information Science and Engineering, Ritsumeikan University

**^c^** Research Institute, Shiga Medical Center

**^d^** Biomedical Innovation Center, Shiga University of Medical Science

**^e^** Department of Health Science, Kyoto Koka Women’s University

Corresponding Author: Akihiko Shiino, M.D., Ph.D.

Division of Biomedical MR Science,

Molecular Neuroscience Research Center,

Shiga University of Medical Science

Seta, Ohtsu, Shiga, 520-2192, Japan

TEL:+81-77-548-2943

FAX:+81-77-548-2943

E-mail: [shiino@belle.shiga-med.ac.jp](mailto:shiino@belle.shiga-med.ac..jp)

Supplemental Figure 1


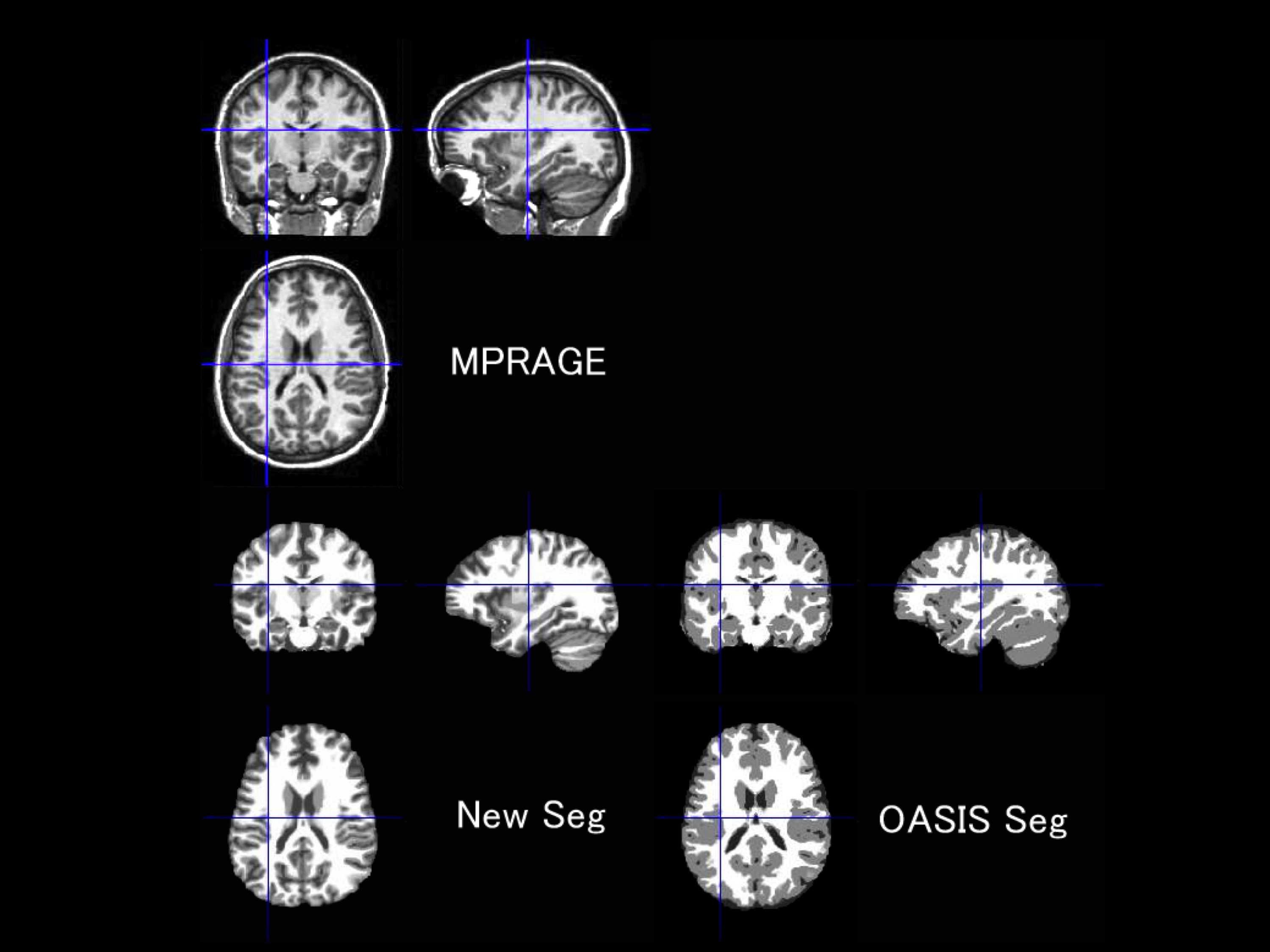


Supplemental Figure 2


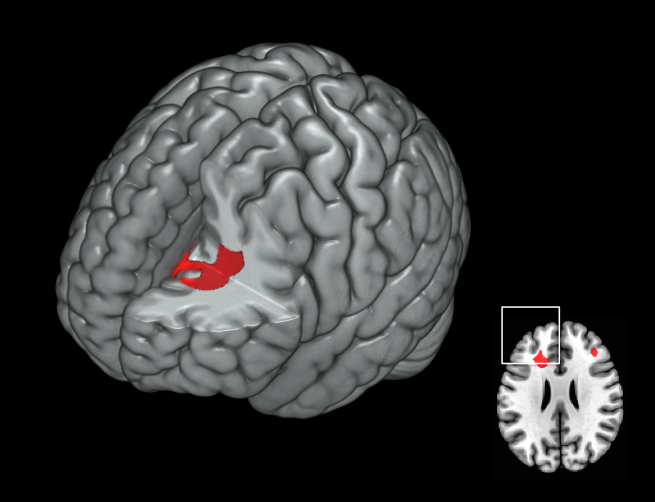


Supplemental Figure 1. Illustration for comparison between the results of segmentation implemented by the “new segmentation” mounted on statistical parametrical mapping (SPM) 8, and segmented example images from the OASIS database (Subject ID; OAS1_0004). As shown in this picture, segmentation accuracy is improved by the “new segmentation” algorithm.

Supplemental Figure 2. Three-dimensional surface rendered image showing the cluster of significance in the left frontal lobe (FWE, p< 0.05, TFCE). Red color indicates larger white matter volume in women than men, showing that the white matter close to Broca’s area is larger in women. The subjects were the total intracranial volume (TIV)-matched 37 man-woman pairs obtained from the OASIS database. All subjects were right-handed.
